# Stigmatization among methadone maintenance treatment patients in mountainous areas in northern Vietnam

**DOI:** 10.1186/s12954-016-0127-9

**Published:** 2017-01-06

**Authors:** Hung Van Nguyen, Huong Lan Thi Nguyen, Hue Thi Mai, Hai Quan Le, Bach Xuan Tran, Canh Dinh Hoang, Huong Thi Le, Cuong Tat Nguyen, Tho Dinh Tran, Carl A. Latkin, Thuc Minh Thi Vu

**Affiliations:** 1 Authority of HIV/AIDS Control, Ministry of Health, Hanoi, Vietnam; 2 Institute for Global Health Innovations, Duy Tan University, Da Nang, Vietnam; 3 Tuyen Quang Provincial AIDS Prevention Center, Tuyen Quang, Vietnam; 4 Institute for Preventive Medicine and Public Health, Hanoi Medical University, Hanoi, Vietnam; 5 Johns Hopkins Bloomberg School of Public Health, Baltimore, MD USA; 6 Department of Hepatobiliary Surgery, Vietnam-Germany Hospital, Hanoi, Vietnam; 7 Department of Immunology and Allergy, National Otolaryngology Hospital, Hanoi, Vietnam

**Keywords:** Stigma, Discrimination, Methadone maintenance treatment, Mountainous area, Vietnam

## Abstract

**Background:**

Stigma and discrimination may adversely affect the benefits of methadone maintenance treatment (MMT) for drug users, especially in disadvantaged settings. This study assessed stigma and discrimination against MMT patients in the mountainous and rural areas in Vietnam and explored their associated factors to inform implementation strategies.

**Methods:**

We interviewed 241 MMT patients in two clinics: one in Tuyen Quang Province’s inner city and the other in Son Duong District, to assess stigma and discrimination that patients perceived and experienced. Socioeconomic status, health behaviors, health status, and history of drug abuse were examined. Multivariate linear and logistic regression models were used to explore factors associated with stigma and discrimination.

**Results:**

The majority of respondents reported experiencing stigma and discrimination including blame/judgment (95.1%), shame (95.1%), disclosure (71.4%), and the fear of human immunodeficiency virus (HIV) transmission by others (74.1%). Unemployed patients were more likely to experience discrimination (Coef = −1.18, 95% CI = −1.87; −0.89). Those who were taking an antiretroviral were more likely to disclose their health status (Coef = 2.27, 95% CI = 0.6; 3.94). In addition, a higher likelihood of being blamed/judged and shamed was associated with those who suffered from anxiety/depression (Coef = 1.59, 95% CI = 0.24; 2.93 and Coef = 1.07, 95% CI = 0.36; 1.79, respectively).

**Conclusions:**

MMT patients in these mountainous areas perceived high levels of stigma and discrimination which were associated with mental health disorders, unemployment, and HIV infection. These findings highlighted the importance of reducing drug use and HIV-related stigma against high-risk populations. Besides, psychosocial and familial supports, as well as job referrals, also play crucial roles in terms of promoting quality of life among MMT patients.

## Background

Illicit drug misuse is a major global public health issue. People who inject drugs (PWID) are more vulnerable to various factors including the transmission of infectious diseases [1]. In 2015, approximately 65% of all people living with HIV were PWID [2]. A study carried out in northern Vietnam also revealed significantly high prevalence of HIV, hepatitis B virus (HBV), and hepatitis C virus (HCV) among PWID (42.4, 80.9, and 74.1%, respectively) [3].

In response, Vietnam has shown vigorous efforts to reduce the number of drug users as well as the incidence of drug injection-related blood-borne diseases at both national and international levels. The methadone maintenance treatment (MMT) program was first introduced in Vietnam in 1996–1997, and by 2012, 61 MMT clinics were available, providing treatment for 20 cities and provinces across the country [4]. In 2006, harm reduction programs were implemented in 21 provinces with various interventions including providing sterile syringes and condoms, changing behavior, and counseling [5]. Vietnam has committed that, by 2020, 90% HIV-positive people know their status, 90% of those diagnosed with HIV engage in antiretroviral therapy (ART), and 90% of ART patients have durable viral suppression (“90-90-90” targets) [6].

Harm reduction services have shown promising results. For instance, the previous literature revealed that MMT could promote the quality of life, reduce the frequency of illicit drug use [7], and minimize HIV-related risk behaviors among illicit drug users [7–10]. Moreover, a needle exchange program (NEP) was effective in reducing blood-borne diseases [11].

However, these programs face formidable challenges partly due to high levels of stigma associated with drug use [12]. These hamper the effectiveness of drug treatment [13], limit the access to drug and HIV/AIDS treatment [14], negatively affect health status, and reduce treatment adherence [15, 16]. Therefore, to maximize the effectiveness of harm reduction services, attention should be drawn to the prevalence of stigma and discrimination experience by drug users.

Stigma and discrimination against most-at-risk populations have been studied in different health care settings in Vietnam [17–19]; very few studies, however, have drawn much attention to rural and mountainous areas where HIV has been increasingly prevalent [20]. This study, therefore, aims to examine stigma among MMT patients in mountainous areas in northern Vietnam.

## Methods

### Study setting and sampling method

We conducted a cross-sectional study from May to August 2016 in Tuyen Quang Province. Two MMT clinics were involved in the study: one in Tuyen Quang City, a third-grade urban area, and the other in Son Duong District, a rural area. In fact, there are three MMT centers in Tuyen Quang Province; however, we excluded one, Yen Son District, because only nine patients have been treated in this clinic due to its new establishment. Methadone treatment in the two clinics is free of charge and funded by the Australian government and the Royal Dutch government. Apart from daily methadone treatment, patients also receive health counseling from health staffs working at these sites.

The sample included patients taking MMT in the selected sites. The eligibility criteria included (1) taking or initiating MMT in the selected sites, (2) presenting at the clinics during the study period, (3) being 18 years old or above, (4) having the capacity to answer the questionnaire, and (5) agreeing to participate. We approached all patients who were at the clinics and introduced the study. Those who met the eligibility criteria were invited to enroll and gave written informed consent if they agreed to participate upon the understanding of the study’s purposes. Interviews were administered in a private room. The patients did not receive any compensation given that the medication was provided free of charge. A convenient sample of 241 patients was recruited in the study. The response rate was 80–90% across the two sites.

### Measurements and instruments

Face-to-face interviews were conducted by well-trained interviewers including health staffs working at the Center for Preventive Medicine, Tuyen Quang Province, and master’s students of public health at Hanoi Medical University.

### Socioeconomic characteristics

Socioeconomic characteristics included age, gender, education level, marital status, employment status, ethnicity, religion, and household monthly income. Household monthly spending was measured with two elements: recurring/regular expenditure (namely food, clothes, rent, utility, education) in the last 30 days and non-recurring/irregular expenditure (e.g., health care, furniture, occasions, travel, construction) in the last 12 months [21, 22].

### Health status

Health-related quality of life (HRQOL) was measured by using the EuroQol-five dimensions-five levels (EQ-5D-5L) instrument. The instrument comprises five domains: mobility, self-care, usual activities, pain/discomfort, and anxiety/depression, with five levels of response: no problems, slight problems, moderate problems, severe problems, and extreme problems. To compute those indexes, we employed the interim scoring for EQ-5D-5L from the cross-walk value set of Thailand due to the unavailability of the Vietnamese population’s preference [23, 24]. The Vietnamese version of EQ-5D-5L was translated and adopted as well as validated elsewhere [24, 25]. Furthermore, we also asked the patients about HIV status and acute and chronic illness.

### Illicit drug use and rehabilitation

We collected the following information on the history of drug abuse and current drug abuse.

### Stigma/discrimination

We referred to the Substance Abuse Self-Stigma Scale by Luoma et al. and measures of HIV-related stigma [26]. In addition, we adapted the conceptual framework by Parker and Aggleton to construct the measure of drug addiction-related stigma among MMT patients [27]. We then piloted the measures in drug users and people living with HIV/AIDS. The final measure of stigma included five dimensions: (1) blame/judgment, (2) shame, (3) discrimination in various settings (workplace, health care services, family, and community), (4) disclosure of addiction or health status (including HIV-positive if seropositive), and (5) others’ fear of HIV transmission among those patients who self-reported being HIV-positive [28]. The respondents were asked if they had experienced any of the above types of stigma within the last month with the response options ranging from 0 to 10 points, indicating “never experience” and “always experience,” respectively. We divided the score into three levels: 0 indicates “never experience,” 1 to 9 suggests “experience,” and 10 indicates “always experience.” The following are examples of the items included:

1. Have you recently been blamed or criticized because of your health status and drug use behaviors?
2. Do you currently feel shame because of your health status and drug use behaviors?
3. Have you felt discriminated against or treated badly by others? In which circumstances (workplace/all health facilities/family/community/others)?
4. Have you ever disclosed your health status and drug use behaviors with others? With whom did you share?
5. (For HIV positives) Has anyone expressed fear of contracting HIV from casual contacts with you?

### Statistical analysis

Data was analyzed by using STATA software version 12.0 (StataCorp LP, College Station, TX, USA). The Mann-Whitney and chi-squared tests were employed to measure the difference in levels of discrimination/stigma between the two study sites. A *p* value <0.05 was considered statistically significant. In this study, a stepwise backward selection strategy was applied along with multivariate Tobit regression to have reduced models. This strategy uses a threshold with the log-likelihood ratio test to have predictors with *p* values <0.2 included.

## Results

Table 1 provides the demographic information of the respondents. In total, 241 patients participated in this study; 167 patients were receiving MMT in Tuyen Quang, and 74 patients were receiving MMT in Son Duong. The most common ethnic group was Kinh (92.2%). The mean age was 40.4 years (SD = 7.49). The majority lived with a spouse or partner (62.3%). The percentage of patients who were self-employed was 47.5% and only 6.4% was unemployed.

**Table 1:** Demographics of respondents

Characteristics	Number	Percent
Education		
<High school	111	47.0
≥High school	125	53.0
Marital status		
Single	54	22.9
Lives with a spouse/partner	147	62.3
Divorced/separated/widow	35	14.8
Employment		
Unemployed	15	6.4
Self-employed	112	47.5
Others	109	46.2
Religion		
No	220	93.6
Others	15	6.4
Ethnicity		
Kinh	213	92.2
Others	18	7.8
Location		
Tuyen Quang	167	69.3
Son Duong	74	30.7
	Mean	SD
Age	40.4	7.49

Table 2 illustrates the health status and risk behavior among the respondents. There were a large proportion of patients who were smokers (75.7%). A small percentage of patients reported health-related impairment: mobility (14%), self-care (10.6%), usual activities (14.4%), pain/discomfort (19.9%), and anxiety/depression (25.9%). The average age at first drug use was 24.4 years (SD = 7.64) and at first drug injection was 29.2 years (SD = 8.72). In addition, the majority was negative for HIV (73.6%) and 25% of the respondents was positive. The percentage of patients who were enrolled in ART was 22.8%. The number of drug rehabilitation was 1.8 (SD = 1.08), and the duration of MMT utilization was 15.3 months on average (SD = 8.66).

**Table 2:** Health status and risk behavior among respondents

Characteristics	Number	Percent
Concurrent drug use	31	13.4
Having a problem in mobility	33	14.0
Having a problem in self-care	25	10.6
Having a problem in usual activities	34	14.4
Having pain/discomfort	47	19.9
Having anxiety/depression	61	25.9
HIV test results		
Negative	170	73.6
Positive	59	25.5
N/A	2	0.9
Receiving ART		
Yes	53	22.8
No	180	77.3
	Mean	SD
Age at first drug use	24.4	7.64
Months of drug use	168.4	83.82
Age at first drug injection	29.2	8.72
Number of drug rehabilitation	1.8	1.08
Duration of MMT (months)	15.3	8.66
BMI	21.2	2.33
EQ-5D-5L index	0.9	0.20
EQ VAS	81.8	15.27

In Table 3, the proportion of stigma and discrimination among MMT patients is shown. We found that most of the enrolled patients experienced several types of discrimination and stigma including blame/judgment (95.1%), shame (95.1%), disclosure (71.4%), and the fear of HIV transmission by others (74.1%). These percentages were higher in Tuyen Quang than in Son Duong, except for the category others’ fear of HIV (*p* < 0.05).

**Table 3:** Discrimination/stigma among respondents

Characteristics	Tuyen Quang		Son Duong		Total		*p* value
	*n*	%	*n*	%	*n*	%	
Blame/judge							
No experience	2	1.2	10	13.5	12	5.0	<0.01
Experience	165	98.8	60	81.1	225	93.4	
Always experience	0	0.0	4	5.4	4	1.7	
Shame							
No experience	2	1.2	10	13.5	12	5.0	<0.01
Experience	163	97.6	61	82.4	224	93.0	
Always experience	2	1.2	3	4.1	5	2.1	
Discrimination							
No experience	7	4.2	7	9.5	14	5.8	0.22
Experience	159	95.2	66	89.2	225	93.4	
Always experience	1	0.6	1	1.4	2	0.8	
Disclosure							
No experience	43	25.8	26	35.1	69	28.6	<0.01
Experience	123	73.7	37	50.0	160	66.4	
Always experience	1	0.6	11	14.9	12	5.0	
Others’ fear of HIV							
No experience	6	50.0	0	0.0	6	26.1	0.01
Experience	6	50.0	9	81.8	15	65.2	
Always experience	0	0.0	2	18.2	2	8.7	
	Mean	SD	Mean	SD	Mean	SD	*p* value
Blame/judge	2.0	1.60	2.8	2.87	2.3	2.11	0.52
Shame	2.1	1.77	3.1	2.96	2.4	2.24	0.24
Discrimination	1.9	1.64	2.7	2.24	2.2	1.87	0.03
Disclosure	1.6	1.70	3.5	3.65	2.1	2.62	0.02
Others’ fear of HIV	2.2	2.48	2.9	3.56	2.5	3.00	0.38

Table 4 indicates the various factors associated with discrimination and stigma against drug users. Blame was positively associated with those who attended high school (Coef = 0.59; 95% CI = 0.03; 1.16). Those unemployed were more likely to be discriminated (Coef = −1.18, 95% CI = −1.87; −0.89). In addition, those who received ARV treatment were associated with a lower likelihood of being blamed and judged (Coef = −0.79, 95% CI = −1.4; −0.12), and they were more likely to disclose their health status and drug use behavior (Coef = 2.27, 95% CI = 0.6; 3.94). Lastly, those who suffered from anxiety/depression were more likely to be blamed/judged (Coef = 1.59, 95% CI = 0.24; 2.93) and shamed (Coef = 1.07, 95% CI = 0.36; 1.79).

**Table 4:** Factor associated with discrimination and stigma among respondents

Characteristics	Blame/judge		Shame		Discrimination		Disclosure	
	Coef	95% CI	Coef	95% CI	Coef	95% CI	Coef	95% CI
Education (vs <high school)								
High school	0.59**	0.03; 1.16	0.58*	−0.04; 1.20			−0.78**	−1.48; −0.08
>High school							−1.46*	−3.01; 0.1
Employment (vs unemployment)								
Worker/farmer					−1.18***	−1.87; −0.49		
HIV test results (vs negative)								
Positive			0.69*	−0.02; 1.40			1.36	−0.27; 2.99
Marital (vs single)								
Divorced/widow					−0.53	−1.21; 0.15		
Income quintile (vs poorest)								
Middle	−0.73**	−1.41; −0.04						
Richest	−0.90**	−1.68; −0.11			−0.77**	−1.41; −0.13		
Number of drug rehabilitation (vs none)								
1 time	−0.46	−1.07; 0.14			−0.47*	−0.99; 0.04		
>2 times							0.55	−0.19; 1.28
Receiving ART (yes vs no)	−0.79**	−1.45; −0.12					2.27***	0.6; 3.94
Location (Son Duong vs Tuyen Quang)	0.78**	0.14; 1.42	1.01***	0.31; 1.71	1.40***	0.81; 2.00	2.16***	1.38; 2.93
Having a problem in usual activities (yes vs no)	1.68**	0.29; 3.07			1.69***	0.53; 2.86	1.19**	0.02; 2.36
Pain/discomfort (yes vs no)							1.11	−0.23; 2.46
Anxiety/depression (yes vs no)	1.59**	0.24; 2.93	1.07***	0.36; 1.79	1.49**	0.36; 2.63	0.77	−0.43; 1.97
Concurrent drug use (yes vs no)							0.79	−0.17; 1.75
EQ-5D-5L index	3.47*	−0.36; 7.3			2.69*	−0.52; 5.90		
Age							−0.05**	−0.1; −0.01

***p < 0.01, **p < 0.05, *p < 0.1

## Discussion

This research enriched the existing literature on MMT-related stigma and discrimination among those who live in rural and mountainous areas in Vietnam by identifying factors related to stigma and discrimination against MMT patients, which have not been well documented in previous studies. Although MMT has been known for reducing risk behaviors and increasing the quality of life among drug users [29–32], our results indicated that stigma/discrimination significantly impacts MMT patients in northern Vietnam and feelings of stigma/discrimination were especially high among unemployed patients. In addition, those who were on antiretroviral treatment were more likely to disclose their health status and participants reporting anxiety and depression were also more likely to report feeling blamed/judged and shamed.

### Stigma against drug users

Stigma and discrimination among drug users have been previously reported in various health care services [17, 19, 33]. In this study, the enrolled patients experienced different kinds of stigma including blame/judgment (95.1%), shame (95.1%), and others’ fear of HIV transmission (74.1%), which is consistent with a study by Jimeez et al. The study showed that 80% of patients reported some level of felt stigma [34]. However, our results are higher than those of a previous study in Hanoi, and Nam Dinh, which reported 17.2% (blame/judgment), 19.9% (shame), and 17.1% (others’ fear of HIV transmission) [17]. These inconsistent results can be explained by differences in the level of awareness about and attitudes towards HIV/AIDS among community members. A previous study by Alemu et al. showed that the severity of stigma and discrimination is influenced by the level of the community’s awareness about HIV/AIDS [35]. Those who live in cities are more likely to be well aware of HIV/AIDS than those who live in rural areas. All the participants in our study live in rural areas, which may help to explain the higher proportion of stigma/discrimination against drug users than that of a study in Vietnam’s capital, Hanoi, and Nam Dinh Province.

Discrimination has been shown to adversely affect the health of illicit drug users. Some previous studies suggested that discrimination and alienation are associated with poor mental and physical health [36, 37]. Our finding remarkably contributed to the literature as we found that those who suffered from mental health problems were more likely to report some forms of stigma and discrimination as well. Further research should be performed to understand the mutual relationship between stigma/discrimination and health among marginalized populations.

### Factors related to stigma against drug users

Our study found that those who were unemployed were more likely to report self-stigmatization, which is consistent with the aforementioned study in Hanoi and Nam Dinh Province. The relationship between stigma/discrimination and employment is a complex process; stigma/discrimination can act as a barrier to work, and being unemployed may fuel stigma against drug users. A review of the literature by Sutton et al. identified that one of the main barriers to work among drug users was the problem of stigma [38]. In Vietnam, stigma and discrimination against people living with HIV/AIDS are a pressing issue due to the propaganda associating HIV with the social evils of sex work and drug use [18, 39–41]; these barriers may prevent them from obtaining employment [42]. Our study was carried out in rural areas, where stigma against drug users is far worse, which may result in the significantly inverse correlation between stigma and employment.

It was also noteworthy to find that those who were on antiretroviral treatment were more likely to report self-disclosure. Evidence suggested that stigma and discrimination against people living with HIV in the community and health care settings persistently hinder the utilization of HIV-related treatment services [18, 41]; this helps to explain why those who were on antiretroviral treatment tended to disclose their health status as they may feel less self-stigmatized. A study by Duong et al. suggested that self-disclosure may help those high-risk populations to increase access to HIV-related care [42].

### Implications

Several implications can be drawn from this study. First, as stigma was higher in unemployed MMT patients, it is suggested that more attention should be paid to the lower socioeconomic methadone group. This can be done by moving MMT clinics to commune health centers so that patients can easily access drug treatment without long-hour traveling, thereby saving a great deal of time and boosting chances of employment. Besides, job opportunities should also be considered by relevant stakeholders. Second, as MMT patients in mountainous areas perceived an unexpectedly high level of stigma and discrimination, mass community-based behavioral change campaigns should be prioritized in order to raise knowledge and awareness about HIV/AIDS. In fact, many Vietnamese hesitate to make contact with HIV-positive people due to the misconception about how HIV is transmitted; a vast majority of people believe that HIV can be easily transmitted by mere touching [43]. Finally, our finding indicated that stigma and discrimination are in an intimate relationship with impaired physical and mental health; therefore, a comprehensive approach combining social, family, and health care supports should be considered to alleviate drug addiction-related stigma, thereby promoting the overall health status of methadone patients.

### Limitations

Several limitations of this study should be taken into consideration. First, our data was cross-sectional; therefore, we could only observe stigma and discrimination prevalence among methadone patients at one point in time. The outcomes of interest, namely stigma and discrimination, were evaluated in the last 30 days, while the health-related quality of life was measured using EQ-5D-5L during presentation at the clinics. This may lead to a temporal relationship between the quality of life and stigma/discrimination. Therefore, a longitudinal study will provide a greater insight into how multiple factors can influence stigma and discrimination against MMT patients. Second, recall bias may occur due to self-reported information, which can lead to data bias. Finally, we chose the convenient sampling technique in our study. This may increase the risk of data bias, which clouds the study results. In addition, this sample included only those patients who had engaged in or accessed MMT services while lacked other drug users without treatment. Thus, these findings are not generalizable to injection drug users not engaged in methadone treatment or non-opioid-injecting participants. Ideally, accessing MMT services may reduce stigma and discrimination by counseling or motivational interviewing, as well as the familial supports for the patients as required by the program. As a result, this might lead to an underestimation of the outcomes of interest.

## Conclusions

In conclusion, our study emphasized that MMT patients in mountainous areas in northern Vietnam experienced significantly high levels of stigma and discrimination. The findings suggested the need for raising awareness among populations about HIV/AIDS to reduce stigma. Moreover, the study also highlighted the importance of social supports, as well as job referrals, in terms of diminishing self-stigmatization and promoting quality of life among MMT patients.

## Abbreviations

AIDS: Acquired immune deficiency syndrome
ART: Antiretroviral therapy
EQ-5D-5L: EuroQol-five dimensions-five levels
HBV: Hepatitis B virus
HCV: Hepatitis C virus
HIV: Human immunodeficiency virus
HRQOL: Health-related quality of life
IRB: Institutional review board
MMT: Methadone maintenance treatment
NEP: Needle exchange program
PWID: People who inject drugs
VAS: Visual analogue scale
